# Experimental Study on the Manufacturing of Functional Paper with Modified by N-Methylmorpholine-N-oxide Surfaces

**DOI:** 10.3390/polym15030692

**Published:** 2023-01-30

**Authors:** Nikolay V. Khomutinnikov, Igor O. Govyazin, Gennady E. Ivanov, Elena M. Fedorova, Igor S. Makarov, Markel I. Vinogradov, Valery G. Kulichikhin

**Affiliations:** 1 Joint Stock Company «Goznak», 17 Mytnaya Str., 115162 Moscow, Russia; 2 A.V. Topchiev Institute of Petrochemical Synthesis, Russian Academy of Sciences, 29 Leninsky Prosp., 119991 Moscow, Russia

**Keywords:** paper, cellulose, N-methylmorpholine-N-oxide, air permeability, strength

## Abstract

The manufacturing of paper with new functional properties is a current problem today. A method of modifying the surface layer of paper by the partial dissolution of cellulose on its surface is proposed. N-Methylmorpholine-N-oxide (NMMO) is proposed for use as a solvent, the regeneration of which provides an environmentally friendly process. It was shown that among the possible hydrate forms of the solvent, the monohydrate and higher-melting forms are optimal for modifying the paper surface. The temperature–time modes of processing were revealed and the weight gain and density increase in the course of modification were estimated. The structural and morphological features of the original and modified paper were studied by X-ray imaging and scanning microscopy. The NMMO surface treatment makes it possible to vary the air permeability of the paper, making it practically non-permeable. The capillary and pore system were radically transformed after the partial dissolution of cellulose and its coagulation, as the formed cellulose film isolates them, which leads to a decrease in surface absorbency. The processing conditions allowing for the optimization of the optical and strength properties of the modified paper samples are revealed. The resulting paper with a modified N-methylmorpholine-N-oxide surface layer can be used for printing valuable documents.

## 1. Introduction

Lignocellulosic biomass is one of the most accessible sources of raw materials [1]. The main components of lignocellulosic mass are cellulose (9–80%), hemicelluloses (10–50%) (included in the list of the most common organic compounds and second only to cellulose) (HC), lignin (5–35%), extractives, and inorganic substances (1–2 wt.%) [2]. The ratio of these components depends on the source of pulp containing cellulose, its place of growth, climatic conditions, etc. [3]. The most interesting product in lignocellulosic pulp is cellulose. Cellulose is a polysaccharide consisting of anhydro-D-glucopyranose units. The glucopyranose units are connected by β-glycosidic bonds. The average polymerization of cellulose depends on the polymer source, for example, for wood, it varies from 5 to 10 thousand; for flax, it reaches 8000; for cotton, the values are up to 20,000. Cotton is distinguished not only by high values of cellulose polymerization degree but also by its content in culture of 95–97% [4]; for flax, depending on the plant part, the cellulose content reaches 92% [5,6]. For perennial plants, the proportion of cellulose can vary in the range of 40–50% [7,8].

In order to obtain technical cellulose, ground plant material is subjected to chemical treatment at elevated temperature and pressure (pulping). Varying the conditions of the mechanochemical treatment of lignocellulosic pulp during the pulping process affects the yield of the cellulose product and its chemical composition [9]. Cellulose (containing some hemicellulose and lignin) is mainly used for the production of paper and cardboard.

Hemicelluloses include xylans, glucans, galactans, mannans, etc. The distinguishing feature of HC from cellulose is the presence of functional groups such as acetoxy groups, carboxyl groups, methoxyl groups, etc. This leads to the fact that HC is insoluble in weakly polar organic solvents such as methanol, ethanol, etc. The most sought-after direct solvents of HC are water and alkalis [10].

The retention of hemicelluloses in the cellulose during the pulping process has a positive effect on the strength properties of the paper, the quality of printing on it, etc. [11]. On the other hand, a certain amount of hemicelluloses not only gives the paper-base certain adhesive properties necessary for the surface bonding of fibers, but also provides the fibrillation of fibers without excessive reduction of their length during grinding. In contrast, the presence of lignin is undesirable in cellulose because it is more vulnerable to oxidation by air oxygen and can play the role of an oxidation catalyst (source of radicals) [12]. However, it has recently been shown that the presence of lignin can have a positive effect on polymer stability, where it acts as an antioxidant in the system [13]. Hence, the “ideal” method of pulping and delignification should ensure the removal of the required amount of lignin and the retention of hemicellulose.

Currently, a large number of pulping options are proposed in the literature that allow for obtaining both alpha enriched (high molecular weight cellulose insoluble in 17.5% alkaline solution) and cellulose products containing dozens of percent of hemicelluloses or lignin [14,15]. The presence of hemicellulose in cellulose is often welcome in the manufacturing processes of paper and board. Hemicellulose plays the role as a kind of binder in cellulose and allows for an increase in the mechanical characteristics of paper [16]. However, we must not forget that the thermal behavior of hemicelluloses is very different from cellulose, as their thermal degradation begins at lower temperatures compared to cellulose [17]. Hence, the paper produced from raw materials with a high hemicellulose content can lose its mechanical and functional properties when exposed to temperature.

According to the Obermans hypothesis and the works of S.P. Papkov, cellulose always contains a certain amount of low molecular weight compounds. Hemicellulose belongs to such compounds. They are responsible for the formation of “horn-shaped” formations in cellulose [18,19,20]. Thus, even cooked cellulose may contain some fraction of heteropolymers and low molecular weight compounds.

The morphology of the cellulose raw material deserves special attention as it can contribute to the characteristics of the output paper. The formation of the required morphology can be achieved by using fibers of known shape and size, controlling the content of hemicelluloses and inorganic compounds, the prehistory of the cellulose, etc. If cellulose is subjected to a physicochemical pretreatment, the specific surface area may change and also redistribute low molecular weight compounds (e.g., redeposition on the fiber surface) [21].

The relative ease of paper manufacturing, the extensive and constantly renewable raw material base of cellulose, and continuous scientific research are expanding the possibilities of paper use [22]. Due to its availability and functional properties, paper has found its application in various fields of human activity from biomedicine and space to the food industry [23]. It is important to note the scalability of most paper manufacturing and processing methods.

The unique system of inter- and intramolecular hydrogen bonds in cellulose is responsible for its chemical characteristics such as high resistance to a number of traditional solvents, packing density, and structure [24,25,26]. As a polysaccharide, cellulose is characterized by excellent hydrophilicity, biocompatibility, biodegradability, and high mechanical properties [27].

At the present time, a large number of methods of the physical and chemical modification of pulp and paper materials has been developed, among them the application of polymer coatings, adhesives, paraffins, and bitumens and the effect of plasma, temperature, etc. These methods of paper modification allow us to extend its functional characteristics and find new areas of application [28,29,30,31,32], shown, in particular, to improve the barrier properties of paper in relation to liquid media, air permeability, mechanical characteristics, etc. In the patent [33] for the creation of contamination-resistant paper for manufacturing banknotes and valuable documents, the application of a coating containing an unpigmented dispersion of aliphatic polyurethane to the surface pre-treated with a solution of polyvinyl alcohol was proposed. In [34], the authors proposed treating at least one side of the banknote with fluorine-containing compounds, which increases the banknote life and protects it from contamination. Another patent [35] describes a method of obtaining non-moldable paper with antimicrobial properties by proposing the application of an antimicrobial coating to the paper containing 1% to 10% by weight of polymerized siloxane and 1% to 10% by weight of a water-soluble fungicide. We can consider the patent in [36] as an example of the use of antibacterial coatings in which isothiazolone-based compounds are used as active ingredients. In the patent in [37], A. Pretsch proposed the application of a film coating (on one or both sides) based on polyolefins or polyethers to the surface of a paper substrate by means of a special device to increase the wear resistance.

The choice of a functional coating is not a trivial task since it must retain the characteristics that ensure the application of typographic elements, provide a barrier function, does not impair the mechanical characteristics of paper, maintain the suitability of paper for processing into banknotes, etc., and also meet the environmental requirements.

An original approach in solving the problem of paper modification is the use of solvents to treat its surface. Unfortunately, the regular nature of the structure of cellulose and the presence of a system of intra- and intermolecular H-bonds exclude its transfer to the fluid state due to heating. Hence, the optimal solution for processing cellulose is to obtain solutions using its direct solvents [38].

Direct cellulose solvents include aqueous solutions of zinc chloride, DMA/LiCl, ionic liquids, N-methylmorpholine-N-oxide (NMMO), etc. [39]. Unlike a number of other direct industrial cellulose solvents, N-methylmorpholine-N-oxide has advantages such as low human and environmental toxicity, high degree of regeneration, high solubility to cellulose and a number of other polymers [40], relatively low price, etc.

NMMO exists in several crystalline forms, and its solubility with respect to cellulose increases with a decreasing water content. The monohydrate form of NMMO with a melting point of 76 °C and water content of 13.3% is widespread. With a water content of about 8%, an increase in the melting point of NMMO (120 °C) and an increase in activity with respect to cellulose can be observed. A further reduction in the water content in the system is inexpedient, since it leads to an even greater increase in the melting temperature as well as to an acceleration of the process of NMMO degradation [41].

In the work in [42], the use of N-methylmorpholine-N-oxide was proposed as a direct solvent of cellulose in order to obtain 3% solutions of cellulose in NMMO for the treatment of paper surfaces and give it new properties. In the patent in [43], they proposed treating fibrous materials (woven or nonwoven) such as paper, felt, or fabric containing predominantly soluble or solvent swollen fibers including wood pulp, cotton, flax, wool, fur, nylon, viscose, polyester and their mixtures with amine oxides to increase the strength of these materials. The strengthening of fibrous materials is due to the swelling or dissolution of fibers and the formation of new physical bonds between them. The amine oxides can be used with additive reagents such as methanol, dimethyl sulfoxide (DMSO), etc. The temperature of the fiber material treatment depends on the type of amine oxide and the cosolvent used. After treating the samples, the solvent is removed with water or another substance active in relation to it. The disadvantages of the invention are the use of cosolvents, the regeneration and separation of which is often a complex and expensive process that involves the presence of several successive stages of material treatment (sample impregnation, its drying, activation of the active reagent by heating the impregnated system, etc.), discreteness (discontinuity) of the process, etc.

Deep interaction of the NMMO with paper can lead to the strong swelling and dissolution of cellulose. Further water treatment of this system results in the removal of the solvent and precipitation of the polymer phase. As a result, a transparent layer is formed at the solvent treatment sites. Paper modified in this way is in demand for food packaging, medical supplies, envelopes, etc. [44]. The advantage of the proposed method is that the modification does not require plasticizers, synthetic polymers, and the used NMMO solvent is almost completely regenerated. Y. Masayuki and Y. Manabu [45] proposed the use of a press and rollers for paper modification. The density of the paper-base varies depending on the desired application, but it was appropriately chosen and used in the range of 30 to 100 g/m^2^. NMMO with a water content of 16% was used to process the paper. The main disadvantages of the invention are the limitations on the density of the paper used and the hydrate form of the solvent used, which does not provide the required activity toward the polymer and requires the removal of excess water, and has a high time and energy consumption of the paper modification process.

Thus, the modification of a paper surface with NMMO is a relevant task. The removal of excess water from the solvent allows for an increase in its activity with respect to cellulose and a reduction in the processing time of the paper substrate. Hence, the aim of this study was to develop a method of paper modification by a solvent with low water content of 8–13%, obtain experimental batches of NMMO-treated paper, and study the functional characteristics of the modified paper.

## 2. Materials and Methods

Two paper options were used in the work as a paper-base:

1. Paper-base made of 75% sulfate bleached softwood cellulose of brand “HB-Extra”, 18% bleached cotton, and 7% polyester fiber of short cut brand “Eslon 1,4 D×6” (fibers length 6 mm) (Sample 1). Sheet thickness of 152 µm, density of 0.72 g/cm^3^. The degree of polymerization of the sulfate bleached softwood cellulose and bleached cotton is 870 and 1170, respectively.
2. Paper-base made of 100% bleached cotton (Sample 2). Paper thickness of 118.2 µm, density of 0.81 g/cm^3^.

The paper was not surface treated (e.g., with polyvinyl alcohol solutions or dispersions based on acrylates, styrene-acrylates, and polyurethanes and other compositions) to preserve the reactivity and surface structure.

For paper modification, direct cellulose solvent N-methylmorpholine-N-oxide (Demochem, Shanghai, China) with a water content of ~8–13.3% (T_melting_~76–120 °C) was used. The water content in NMMO was determined using K. Fischer titration methods (EXPERT-007M, Moscow, Russia) as well as by solvent melting points using polarization microscopy (Boetius, VEB Kombinat Nadema, Ruhla, Germany, former GDR) and differential scanning calorimetry (DSC) (DSC 2920, TA Instruments, New Castle, DE, USA). The measurements were carried out in 70 µL aluminum oxide crucibles in the temperature range from 30 to 200 °C at a heating rate of 10 K/min. The inert gas (argon) flow rate was 10 cm^3^/min. NMMO with the required water content was used to modify the paper substrates. Distilled water at room temperature was used to remove the solvent.

**Figure 1:**
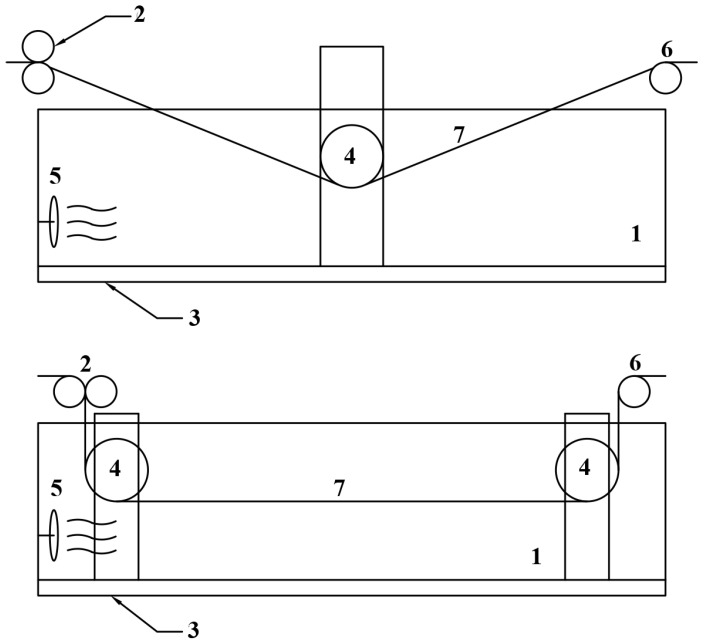
**Schematic diagram of the device for treatment of paper substrate surface:** 1—heated tank (bath) with solvent; 2—pressing rollers; 3—heater; 4—directing rollers; 5—pump for solvent; 6—feeding rollers; 7—cellulose substrate.

The paper was processed by passing the substrate through the molten solvent; the schematic diagram of the device is presented in Figure 1.

To form the modified layers on the surface of the paper, a sheet of cellulose substrate is sent to the pre-filled NMMO bath (1) in which the set temperature is maintained using heaters (3), through the feeding rollers (6) into the container. With the help of guide rollers (4) the sheet of paper is immersed in the solvent and their position determines the path in the bath. After leaving the bath, the excess solvent is removed from the surface with the help of squeeze rollers (2). The processing time of the paper did not exceed 10 s. Then, the sheet goes into the precipitation and rinsing baths with water. Washing is carried out with warm water heated to a temperature of 55–60 °C. The washed paper sheet is dried on drying cylinders and reeled at temperatures of 95–105 °C.

The duration and temperature of the water precipitation bath was selected based on the conducted experiments. According to the assessment of the elemental chemical composition of the paper, under the selected conditions, it was possible to remove all of the NMMO.

The level of solvent in the bath (1) is maintained by the dosing pump (5), into which the NMMO comes from the solvent tank or solvent regeneration system.

The properties of the paper before and after modification of its surface with N-methylmorpholine-N-oxide were determined by standard methods: thickness according to ISO 534:2011 on an automatic micrometer by “Lorentzen & Wettre” (Stockholm, Sweden); folding endurance was evaluated according to the Schopper method according to ISO 5626-78 on an “Ufa device” (Ufa, Russia); water absorbency at one side wetting according to the Cobb method was determined according to ISO 535:1991 with a contact time with water of 60 s on a Cobb Sizing Tester by “Lorentzen & Wettre” (Stockholm, Sweden); breaking strength according to ISO 1924-1:1983 on a L&W SE 062L “Lorentzen & Wettre” dynamometer (Stockholm, Sweden); Elmendorf tear resistance according to ISO 1974:1990 on an Elmendorf device from “TMI”, USA, Model ED 32; air permeability according to ISO 5636-3:1992 on a Bendtsen automatic paper surface roughness and air permeability tester, model K513 from “Buchel B.V.” (Veenendaal, The Netherlands); brightness according to ISO 2470-1:2016; and color characteristics in the CIELab colorimetric system on a L&W SE 071 spectro-colorimeter by “Lorentzen & Wettre” (Stockholm, Sweden).

The structure of the paper was studied by X-ray diffraction analysis (XRD). X-ray diffractometry was performed using a Rigaku Rotaflex RU-200 unit equipped with a rotating copper anode (linear focus 0.5 × 10 mm, source operating mode 50 kV–100 mA, wavelength of the characteristic CuKα λ = 1.542 Å, secondary graphite monochromator), horizontal D-Max/B goniometer, and scintillation detector. X-ray imaging was performed in the “reflection” geometry according to the Bragg–Brentano scheme in the mode of continuous θ–2θ scanning in the angular range of 5–40°, the rate of 2°/min, and the scanning step of 0.04°. The measurements were performed at room temperature.

The surface morphology of the original and modified paper was studied by low-voltage scanning electron microscopy (SEM) on a FEI Scios microscope (Hillsboro, OR, USA) at an accelerating voltage of less than 1 kV in the secondary electron mode.

## 3. Results and Discussion

Paper is universally accessible thanks to its enormous potential for applications such as the collection and storage of energy, biomedicine, packaging, etc. The papermaking technique dates back to the Eastern Han Dynasty in China and is considered as one of the four great inventions. Nevertheless, existing problems in the traditional paper industry have become a concern, along with the development of science and the global circular economy chain. Improving the performance of paper is a major challenge. Changing the functional properties of paper is possible either by adding special substances to the system or by changing the structure and morphology of the substrate. The second method combined with the use of non-hazardous and reclaimed reagents is of great interest.

**Figure 2:**
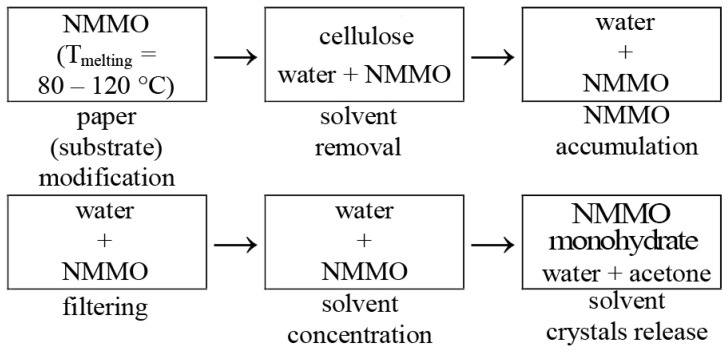
Block diagram of NMMO regeneration.

The current study proposes the use of N-methylmorpholine-N-oxide, which is commercially available for the treatment of paper surfaces. Pre-prepared and characterized NMMO using optical microscopy, DSC, and the K. Fischer method was used to treat the paper surfaces with further regeneration (Figure 2).

Next, we proceed to the stage of the accumulation of the used aqueous NMMO solutions without dwelling on the previously described stages of substrate modification and NMMO removal. When the required NMMO content is reached, the system enters filtration, where low molecular weight components and heteropolymers released during dissolution of the paper substrate surface are removed using membranes and others. Next, the purified aqueous NMMO solution is fed to remove excess water and obtain a concentrated water–solvent system. In the last stage, if necessary, the NMMO monohydrate is deposited with acetone or other non-solvents at a temperature of at least 50 °C. Acetone saturated with water should be regenerated. Technological features and issues related to the regeneration of the solvent used are described in [46,47,48]. The regenerated solvent is used again in the initial stages of paper processing.

**Figure 3:**
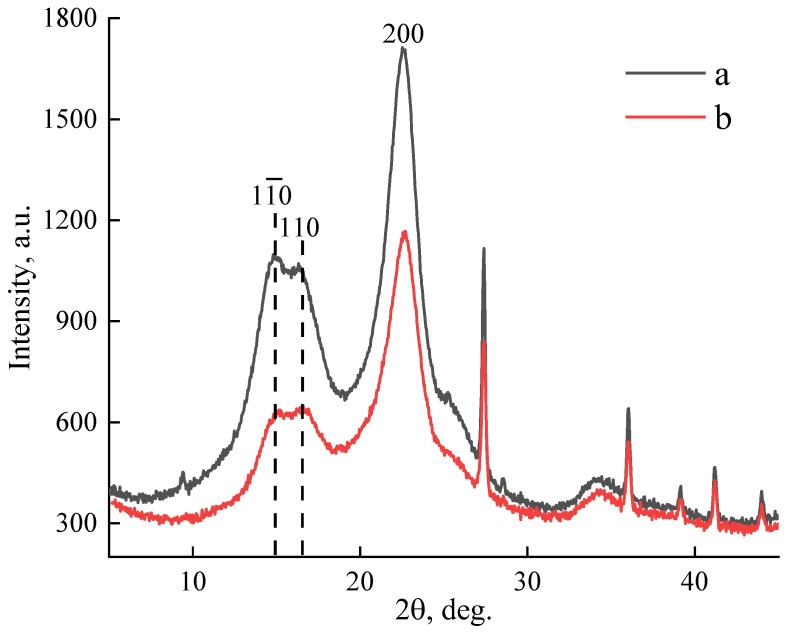
Diffractograms of paper samples before (**a**) and after (**b**) solvent treatment.

The immersion of paper in the solvent will be accompanied by the interaction of cellulose with NMMO, which will directly affect the structure of the resulting substrate (Figure 3).

The native cellulose (polymorph I) from which the paper was obtained was characterized by peaks in the 2𝜃~14.8°, ~16.6°, and ~22.6° regions, which corresponded to planes with Miller indices 1–10, 110, and 200 [49]. The interaction of cellulose with the solvent, for example, in the process of obtaining spinning solutions and the isolation of the polymer phase during coagulation, formed a different structural organization accompanied by a cardinal change in the diffraction pattern, shifting the main peaks in the region of 2 𝜃~12.3°, ~20.5°, and ~21.9° (polymorph II). In the case of short-term treatment of the paper surfaces with solvent, the course of the curves in the diffractograms changed insignificantly (Figure 3). However, the intensity of the peaks changed after paper treatment. For peaks 1–10 and 200 it decreased, while for 110, in contrast, it increased. The ratio of intensities for peaks 1–10 and 200 increased from 1.57 for the original paper to 1.83 for the modified paper. The crystallinity of the original paper was 57.6%, while after NMMO treatment, it decreased to 47.8%. Thus, after a brief interaction of the paper with the solvent, a decrease in the structural ordering of the cellulose was observed.

**Figure 4:**
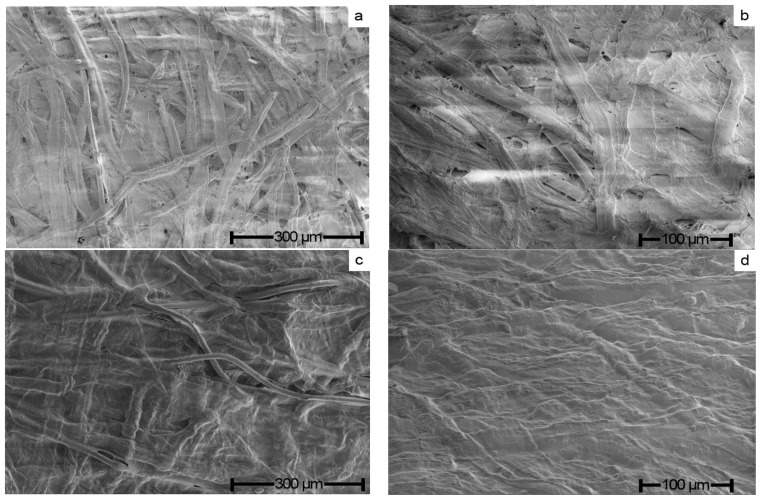
Morphology of the original paper (sample no. 1) (**a**,**b**) and modified with NMMO (**c**,**d**).

Paper is a fibrous material with mineral additives that have a complex capillary-porous structure that depends on the type and characteristics of its constituent fibers. The shape and size of the fibers as well as their mutual spatial arrangement determine the features of the paper structure and its mechanical and deformation characteristics. Figure 4 shows the SEM-microphotographs of the original paper sample no. 1 and the sample exposed to the solvent.

The morphology of the original paper was represented by the traditional random weave of wood, cotton, and polyester fibers. The surface of the paper was not smooth, its profile was significantly complicated by numerous recesses, protrusions, and pores. Cellulose fibers composing the paper had the form of flat strips connected to each other by fibrous “bridges” and film formations most likely consisting of hemicellulose. After forming the paper, polyester fibers retain their cross-section and smooth surface. The morphological picture changed dramatically after the treatment of the paper surface with NMMO. Most of the cellulose fibers that made up the paper lost their shape and were transformed into an uneven monolithic surface. In contrast, the polyester fibers did not interact with the solvent and retained their shape and morphology.

As the paper interacted with the solvent, the resulting solution flowed into the pores, filling them. After removing the solvent with water, the deposited polymer phase remained in the pores in the form of a cellulose film. As a result, after paper modification, its mass (grammage (g/m^2^)) and density will change. The identified changes in mass and density for sample nos. 1 and 2 are presented in Table 1.

As can be seen from the table, for samples treated in NMMO, there was an increase in the mass (density) of paper. Apparently, in the process of the regeneration of dissolved paper surfaces and its further drying, the substrate contracts by changing the structure of the cellulose, forming a different system of hydrogen bonds. With an increase in the time of paper processing, the depth of interaction of the solvent with the cellulose increases and as a result, the values of mass 1 m^2^ and density can reach higher values.

The breaking strength, tear resistance, relative elongation, etc. are among the most important characteristics for paper (Table 2). Information on these mechanical parameters makes it possible to identify the optimum areas of use for the resulting paper.

For papers made from wood cellulose and treated with NMMO, there was a dramatic drop in the tear resistance and breaking strength. The observed decrease in values can be compensated for by introducing cotton and polyester fibers into the wood cellulose. For cotton-based paper, a slight increase in MD tear strength and breaking strength was observed. The relative elongation for the treated paper was increased by almost 40% compared to the original samples.

Thus, it was found that the treatment of paper with a solvent containing NMMO makes it possible to form layers on the surface of the paper, which are similar in their properties to polymer films. The thickness of the formed layer is much thinner compared to the fibrous core of the paper. As a result, the breaking load, etc. acts simultaneously on the thin surface films and on the core with a reduced thickness. It is also important to take into account the fact that the concentration of cellulose in the solution of the boundary layer is insignificant and during the coagulation of the polymer in this layer, numerous defects will form in the form of vacuoles, etc., which then collapse during drying. Hence, the destruction of the modified paper will occur according to a different mechanism compared to the original paper. Coagulation of the modified paper in cold water will promote the formation of stronger films and reduce the deformation properties. Increasing the temperature of the precipitator, in contrast, makes it possible to increase the deformation properties with some loss in the strength values.

The formation of film coatings on paper and paper products has been proposed for use not only to change their mechanical characteristics, but also to control the transport properties. The most interesting transport properties are air permeability and water sorption. In [50], it was shown that the deposition of a polylactide coating on sack paper led to a significant decrease in its porosity due to the formation of a polymer film on the surface of the paper material. The polymer penetrated into the voids between the cellulose fibers and also made the micropore system inaccessible. This led to a reduction in the air permeance and surface absorbency of the sack paper.

The air permeability of cotton paper was 55 mL/min when using wood cellulose, and with PE fibers, it increased to 150 mL/min. After treatment of the paper with the solvent of sample nos. 1 and 2, the air permeability values dropped to zero (Table 3). The reason for this was the partial dissolution of cellulose fibers and the formation of a viscous cellulose solution on the surface of the paper, which closed the pores of the paper, and after washing off the solvent, was transformed into a thin film. A decrease in air permeability to zero indicated an improvement in the barrier properties of the paper.

In addition to the barrier properties, the air permeability of paper allows one to evaluate the application properties of paper during its use, especially if the paper products are subjected to bending, tearing, and other mechanical effects in the process of use. In this case, the paper sample was subjected to an eight times crease process using a method and apparatus specially developed by IGT (Almere, The Netherlands), after which the air permeability of the paper is determined. The crease process destroys the fiber bonds in the paper and in the case of paper with a solid surface coating, it also destroys the integrity of the coating. Therefore, a reduction in the air permeability of the paper after eight times crease is indirectly an indication of an improvement in the integrity of the application properties of paper during its use.

Table 3 shows the change in air permeability of the original sample nos. 1 and 2 as well as the same samples after eight times crease before and after modification of the paper surface with N-methylmorpholine-N-oxide.

Apparently repeated deformation of the samples led to the destruction of the surface film and to the formation of defects in the area consisting of undissolved fibers, resulting in a significant increase in air permeability.

Paper products are hygroscopic, as cellulose fibers easily absorb moisture [51]. In the process of use, paper products constantly exchange moisture with the environment including direct contact with water, which in turn will lead to a loss of mechanical and functional properties, geometric size, and shape of the paper. To eliminate the negative effect of moisture on paper, various methods have been proposed from the introduction of special additives in the paper pulp to the formation of surface films based on hydrophobic materials such as polymers, organosilicon water repellents, etc. Dissolution of the surface layer of cellulose allows the fluid solution to penetrate into the voids and capillaries of the paper. Coagulation of the polymer then forms a film that is identical in chemical composition but has a different structure. The effect of this film on surface absorbency is shown in Table 4.

As we know, the average surface water absorbency of office paper is about 50 g/m². The decrease in the surface absorbency of paper after modification of its surface layers is quite expected—the formation of a cellulose film with a structure different from the original cellulose on the surface of the material creates an obstacle for liquid to spread into the thickness of the paper. As a result, the absorbency to water and oils decreases, that is, the resistance to contamination and wear increases.

Quality paper has a high brightness, which depends on the processing time of the substrate, as found in the current study. To achieve the highest brightness values, the treatment time should not be more than 8 s (determined by the paper pulling speed and the path length in the solvent volume). Then, the solvent was removed with warm water (60 °C) and dried at 100 °C. The resulting brightness values are presented in Table 5.

If the above parameters are maintained, it is possible to obtain paper with a suitable brightness while maintaining good physical and mechanical characteristics. As can be seen from the table, the treatment of wood cellulose-based paper led to a decrease in brightness by almost 10%; for cotton paper, this indicator decreased by 7%. The difference in the observed brightness values of sample nos. 1 and 2 was apparently due to the presence of significant amounts of additives such as lignin and hemicellulose in the wood cellulose, which often concentrate on the paper surface during the dissolution of surface layers and subsequent extraction of the polymer phase (during coagulation).

Opacity is a property of paper that describes the amount of light that passes through it. Paper with lower opacity is more transparent and allows more light to pass through. The opacity of paper determines the extent to which a print on one side of the paper will be visible from the back. The opacity of paper based on wood cellulose decreased from 92.9% to 90.2% after treatment, allowing the modified substrates to be classified as Class C paper. A similar decrease in opacity was observed for cotton paper.

The hue of the paper was evaluated as CIE LAB, model (CIE L*, a*, b*) by the colorimetric method. The color characteristics for the original and treated paper are shown in Table 6.

Modification of the paper surfaces leads to a slight decrease in the L* parameter, which indicates a decrease in luminosity. The content of red and green (parameter a*), in contrast, increased. Parameter b* also increased for the modified paper, which indicates an increase in the content of blue and yellow color. As is known, the degree of polymerization and the alpha-fraction content of cellulose in cotton is higher in comparison with wood cellulose, which makes it more resistant to the action of solvents. Therefore, the observed decrease in color characteristics is more pronounced for samples based on wood cellulose.

Hence, by varying the paper composition and the surface treatment conditions of NMMO paper, its optical properties can be changed. Therefore, a drop in brightness can be as high as 64–68% when the paper is treated with solvent, that is, it is dissolved deeply. High drying temperatures and suboptimal washing temperatures can also lead to a decrease in brightness. The paper opacity can be as high as 94% when the conditions are varied. For offset and metallographic printing methods, the paper showed good printability. Control of the presence of visual elements on these paper samples showed that they were visualized and perceived in full without distortion or deterioration of the properties.

## 4. Conclusions

Thus, a method of modifying the surface of paper samples of various compositions with a direct cellulose solvent N-methylmorpholine-N-oxide, which is almost completely regenerated and used in a closed cycle, was developed and proposed. It was shown that the paper surface treatment can be carried out in continuous and periodic modes with treatment of one or both sides, locally and over the entire surface of the substrate. The optimal concentration ranges of the solvent and temperature–time parameters of the treatment were revealed. It was shown that the use of a solvent with a moisture content of up to 13% makes it possible to modify the paper with the formation of a cellulose film on the surface in short intervals of time. The height of the modified layer can be varied depending on the time of the solvent treatment of the substrate. The formed cellulose film had a different structure and morphology than the original paper. The observed structural changes led to a decrease in the air permeability, water absorption, and optical properties of the paper (brightness and opacity). Varying the processing conditions of the paper substrate makes it possible to achieve the required values of mass, density, and mechanical characteristics, such as increasing the breaking strength and tear resistance.

Replacing the water in the coagulation bath with alcohols or aqueous NMMO solutions in the future opens up new possibilities for modifying the paper and its functional properties.

The resulting paper samples can be used to produce a variety of products including paper for valuable documents.

## Figures and Tables

**Table 1:** Weight and density of paper sample nos. 1 and 2 before and after modification.

	Sample No. 1		Sample No. 2	
	Before	After	Before	After
Grammage, g/m^2^	109.9 ± 3.5	113.3 ± 3.5	95.9 ± 3.0	99.7 ± 3.0
Density, g/cm^3^	0.72 ± 0.05	0.82 ± 0.05	0.81 ± 0.05	0.84 ± 0.05

**Table 2:** Strength properties for paper sample nos. 1 and 2 before and after modification.

	Sample No. 1		Sample No. 2	
	Before	After	Before	After
Tear resistance, mN				
-Machine direction (MD)	1154 ± 300	807 ± 230	1064 ± 230	1136 ± 210
-Cross direction	1737 ± 250	1515 ± 300	1106 ± 240	942 ± 190
Breaking strength (cross direction), number of double bends	2140 ± 200	1250 ± 220	1350 ± 250	1660 ± 350

**Table 3:** Air permeability of paper sample nos. 1 and 2 before and after modification and crease.

	Sample No. 1		Sample No. 2	
	Before	After	Before	After
Bendtsen air permeability of original paper, mL/min	150 ± 40	0.00 ± 0.04	55 ± 25	0.00 ± 0.02
Bendtsen air permeability after 8 times crease, mL/min	1237 ± 250	773 ± 200	383 ± 130	28 ± 10

**Table 4:** Surface absorbency of paper sample nos. 1 and 2 before and after modification.

	Sample No. 1		Sample No. 2	
	Before	After	Before	After
Surface water absorbency at one-side wetting (Cobb_60_), g/m^2^				
-side 1	108 ± 5	29 ± 5	81 ± 6	28 ± 3
-side 2	111 ± 7	29 ± 4	85 ± 4	29 ± 3

**Table 5:** Brightness values of paper sample nos. 1 and 2 before and after modification.

	Sample No. 1		Sample No. 2	
	Before	After	Before	After
Brightness R457, %				
-side 1	83.8 ± 2.5	73.7 ± 2.5	87.6 ± 2.7	80.8 ± 2.3
-side 2	83.7 ± 2.3	74.6 ± 2.4	87.6 ± 2.6	80.6 ± 2.4

**Table 6:** Color characteristics of paper sample nos. 1 and 2 before and after modification.

	Sample No. 1		Sample No. 2	
	Before	After	Before	After
Color characteristics:				
L*				
-side 1	95.46 ± 1.50	92.95 ± 1.38	96.57 ± 1.43	94.70 ± 1.23
-side 2	95.41 ± 1.45	93.09 ± 1.33	96.55 ± 1.38	94.66 ± 1.26
a*				
-side 1	(−0.86) ± 0.25	0.33 ± 0.25	(−0.57) ± 0.25	(−0.24) ± 0.22
-side 2	(−0.87) ± 0.22	0.25 ± 0.22	(−0.58) ± 0.23	(−0.25) ± 0.21
b*				
-side 1	4.19 ± 0.35	7.75 ± 0.29	3.04 ± 0.22	5.00 ± 0.24
-side 2	4.23 ± 0.21	7.29 ± 0.32	3.02 ± 0.23	5.11 ± 0.22

## Data Availability

Not applicable.
